# The relative importance of information items and preferred mode of delivery when disseminating results from trials to participants: A mixed‐methods study

**DOI:** 10.1111/hex.13402

**Published:** 2021-12-08

**Authors:** Jessica Wood, Seonaidh C. Cotton, Katie Gillies

**Affiliations:** ^1^ Health Services Research Unit University of Aberdeen Aberdeen UK

**Keywords:** clinical trials, dissemination, focus groups, interviews, mixed methods, participants, results

## Abstract

**Background:**

Participants want to receive the results of trials that they have participated in. Dissemination practices are disparate, and there is limited guidance available on what information to provide to participants and how to deliver it.

**Objectives:**

This study aimed to establish what trial participants believe should be included in a results summary and how this information should be delivered.

**Methods:**

A mixed‐methods design was used with focus groups and interviews involving women convenience‐sampled from two host randomized‐controlled trials. Participants ranked information items in order of their importance for inclusion in a trial results summary and potential modes of delivery by preference. All participants provided written informed consent.

**Results:**

Sixteen women (mean age [SD] = 71.6 [9.7] years) participated. Participants ranked ‘individual results from the study’ and ‘summary of overall trial results’ as most important. Themes such as reassurance and setting results in context were identified as contributing to participants' decisions around ranking. ‘A thank you for your contribution to the study’ was ranked the least important. Delivery *by post* was the preferred mode of receiving results, with receiving a hard copy of results cited as helpful to refer back to.

**Conclusion:**

Our findings provide insight into what information trial participants deem as important when receiving trial results and how they would like results delivered. Involving patients during development of trial results to be communicated to participants could help to ensure that the right information is delivered in the right way.

**Patient or Public Contribution:**

Public partners were involved in focussed aspects of study conduct.

## 1 BACKGROUND

The importance of disseminating results to people participating in clinical trials has been highlighted in recent years based on participants' requests to receive results, which aligns with efforts to increase transparency in research.^1^, ^2^, ^3^ Current best practice guidance on an optimal approach to trial results dissemination to participants is limited.

A recent study found that the majority of trials (87.7%) intended to report findings to participants.^4^ However, only 18.8% reported an active effort to disseminate results, with the majority of trial teams (80.5%) placing the responsibility on local clinical care teams or leaving the onus on participants themselves to actively locate the results.^4^ A further investigation examining dissemination outcomes in 1818 studies found that only 40% had disseminated results to trial participants (or planned to) up to 2 years after publication.^5^ Various barriers have been reported as to why researchers do not widely disseminate results and include the following: their own perceptions that participants would not be interested in receiving the results and lack of knowledge of how to disseminate results to lay audiences.^2^, ^5^

The Health Research Authority (HRA), who are a unified national system for health research governance in the United Kingdom, published guidance in 2015 on the type of information to provide study participants at the end of study and suggest that this is in the form of a summary form, written in lay language.^6^, ^7^ Although there is guidance in place, there remains a lack of clarity in determining the content and mode of delivery for trial results.

Previous research on trial dissemination is limited, being undertaken largely in one clinical context (oncology‐based trials) or based on hypothetical trial scenarios presented to participants.^8^, ^9^, ^10^, ^11^, ^12^, ^13^, ^14^, ^15^ Additionally, the focus of much of the research to date has been participant comprehension of results rather than exploring what information participants wanted to know or how to deliver this information to them.^10^, ^16^, ^17^

Understanding which information trial participants deem to be important and how best they would like trial results to be disseminated may provide valuable insight for trial teams to better prepare their dissemination plans. The aim of our study was to determine what information participants with lived experience of trial participation believe should be included in a summary of trial results and how this information should be delivered to them.

## 2 METHODS

### 2.1 Study design

The study design was an explanatory mixed‐methods design, in which the data were collected concurrently, but analysed sequentially (i.e., quantitative data analysed first and qualitative data analysed to inform the quantitative findings). This mixed‐methods design was used to allow a more in‐depth understanding of the reasons why women valued certain information items or modes of delivery over others but also to allow more general perspectives about the provision of trial result summaries to be explored.^18^

### 2.2 Participants and consent

Potential participants were convenience‐sampled from two host trials involving female participants (PROSPECT, which had two parallel randomized trials evaluating different surgeries for primary transvaginal anterior or posterior compartment prolapse surgery [ISRCTN60695184],^19^ and VUE, which also comprised of two parallel randomized‐controlled trials of surgical options for upper compartment [uterine or vault] pelvic organ prolapse [ISRCTN86784244]).^20^ Inclusion criteria were previous consent to be contacted about future research and resident within Aberdeen City and Shire to facilitate travel to interviews/focus group. Participants unable to speak English and those unable to provide informed consent were excluded from the study. The PROSPECT participants had received the 2‐year trial results, but the VUE participants had not yet received trial results. An invitation letter and participant information leaflet were sent to eligible PROSPECT and VUE participants by the respective study team, and women could opt into taking part in the study by returning a reply slip or contacting the study team. Reminders were sent approximately 2 weeks following initial invitations. All participants provided written informed consent.

### 2.3 Preparation of resource materials

Existing literature and the HRA guidance^6^ on feedback of trial results to participants were used to inform the topic guides (Appendix 1) for the focus groups and interviews. The topic guide was semi‐structured in format and focused on areas relevant to the study's overarching aims, but was flexible to allow participants to express their opinions and views. The topic guide and prompts were further developed and refined through piloting with colleagues and the research team before the planned focus group discussions. Two ranking exercises to help participants prioritize the information and mode were developed based on the suggested information items and modes of delivery in the HRA guidance.^6^ The information items included information content items (contact details for questions, how to report side effects, information on what happens after the study, invitation for future research, study title and publication reference, summary of individual results, summary of results, summary of study, thank you for participation, treatment received) and items on mode of delivery (DVD, email, in‐person, link to website, post). In advance of participants ranking the information items, they were asked to consider what information they would want to know at the end of a trial. Focus groups and interviews were chosen to provide more in‐depth investigation of information preferences generated in combination with the ranking exercises. The use of focus groups also enabled participants to hear and consider other people's views and preferences.

### 2.4 Data collection

#### 2.4.1 Focus groups and interviews

Focus groups with PROSPECT participants were conducted in March 2018, and interviews with VUE participants were conducted between November and December 2019 at the Heath Services Research Unit, University of Aberdeen. Focus groups were the research team's preferred mode of data collection for this project. However, to maximize participation, from the outset, participants were offered the option to attend an interview if they could not attend a focus group. Due to participant availability, interviews were conducted with participants from the second trial cohort (VUE trial). Focus groups were moderated by the study team (Authors 2 and 3) and interviews were conducted by the lead authors. All participants completed both ranking exercises; in PROSPECT, this was done as an electronic group ranking activity, which allowed an immediate summary of results. Items were ranked from 1 to 10 (1 = *most important*, 10 = *least important*) or 1 to 5 (1 = *most preferred*, 5 = *least preferred*) and also completed individually in the VUE interviews and completed as an electronic group ranking activity in the PROSPECT focus groups. Results from the ranking exercises were used as prompts to further explore perspectives within the focus groups and interview discussions. Focus groups and interviews were audio‐recorded and transcribed verbatim.

### 2.5 Data analysis

#### 2.5.1 Quantitative data analysis

Data from the ranking exercises were analysed using SPSS^21^ to calculate the median and interquartile ranges (IQRs) for information content items, and mode of delivery options. The median, IQR and, if needed, the range were used to determine the order for both ranking exercises by participant group (VUE and PROSPECT). The mean age of the participants and the median length of the interview were also calculated for the two trial participant groups.

#### 2.5.2 Qualitative data analysis

The qualitative data generated were largely used to expand on the results of the ranking exercises. The transcribed manuscripts were compared to the original recordings to correct any discrepancies between the two. Data were analysed using the ‘Framework’ approach.^22^, ^23^ A priori themes were informed by the quantitative data on the most/least important items from the ranking exercise and any additional themes were generated inductively from the interview data. The first four transcripts were analysed using an open coding approach to develop the thematic framework. All coding themes initially identified in the data set by Jessica Wood were reviewed by Katie Gillies and Seonaidh C. Cotton for agreement. Full coding was then completed in NVivO‐12, and data were charted onto a framework matrix.^24^ Comparisons of data within and across the VUE and PROSPECT participant groups were performed and mapped. Quotes were then selected that helped to illustrate the overall groups' reasons for ranking items and to show any discordant views.

### 2.6 Patient public involvement

This project involved contribution from public partners who were already working with a research team on a larger project exploring how to share trial results with participants (RECAP—researchregistry4085). Public partners contributed to focussed activities for this add‐on Masters project. The ranking exercises and patient information leaflet were reviewed by two public representatives for ease of understanding and revised accordingly.

## 3 RESULTS

### 3.1 Participant characteristics

Invitation packs were issued to 72 potential study participants from the PROSPECT trial; 10 agreed, and 2 focus groups were conducted. Fifty‐three invitation packs were issued to potential study participants from the VUE trial, with six agreeing to be interviewed. All study participants were women, with a mean age of 71.6 years (SD = 9.7). The median focus group and interview times are shown in Table 1.

**Table 1:** Participant characteristics

	VUE	PROSPECT
Number of participants	6	10
Age (mean), years	68	73
Age (range), years	44–80	63–83
Focus group/interview median time (min:s)	31:09	59:31

#### 3.1.1 General perspectives on information deemed important in trial results summaries

Before completing the ranking exercises, focus groups and interview participants were invited to suggest what information they deem to be important when receiving trial results. A number of participants suggested that information about implications for the future would be important, specifically in relation to what they could expect longer term. These were split into some participants wanting to know the longer‐term outcomes post trial treatment (i.e., long term follow‐up), with others wanting to know how the trial would influence treatments offered within the NHS. Linked to this was women reporting that they wanted to know the overall ‘success’ of the trial, in her words what the primary outcome was. Others talked about the importance of having a contact person to talk to in case they had any further questions, and also knowing how other women in the trial had ‘got on’.

*So, who is going to read the results, what are the results going to impact on future women, future clinical decisions*. (PROSPECT Participant 1 Focus Group 1)

*Namely how many people it was successful in, and I suppose how many people were in it in the first place, how many people it was successful, that would be an idea*. (VUE Participant 6)

### 3.2 Information content items deemed most important

Overall ranking summaries for the information content are presented in Table 2. There was discordance between the two groups in terms of information content items that were deemed the most important. The PROSPECT participants ranked ‘If you can be given your individual results from the study' as the most important information content item, with the VUE participants ranking it as sixth. Some of the PROSPECT participants voiced poor outcomes following surgery as reasons for wanting to receive individual results and ranked this information as the most important as they felt that the overall 2‐year results that they had received previously did not match their own experiences. In addition, some PROSPECT participants mentioned media attention as possibly contributing to wanting to receive their own individual results and why they ranked this information content item highly.

**Table 2:** Ranking of information content items (most to least important)

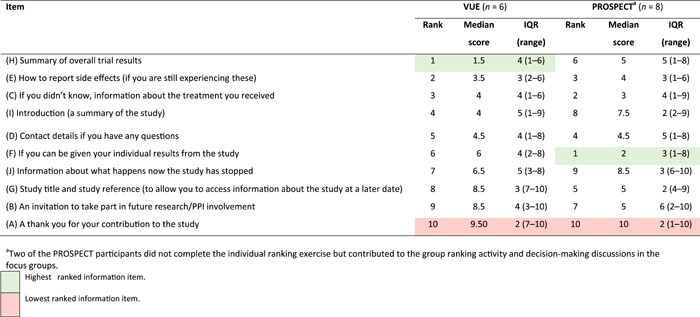

Abbreviation: IQR, interquartile range.

^a^ Two of the PROSPECT participants did not complete the individual ranking exercise, but contributed to the group ranking activity and decision‐making discussions in the focus groups.

[When discussing the overall results in PROSPECT] *‘Obviously we must be an unfortunate lot. I've spoken to different people that had it done, even people I was in the ward with, and there were five of us in at the same time, and out of that five, three of us are still getting problems. …[When asked if it would be helpful to have other information about how you as an individual fit in] …Probably, because as a main group, as we were saying, the majority thought it had helped. Well, I'm only going by the people I've spoken to’*. (PROSPECT Participant 3 focus group 2)

*That [receiving individual results] would have meant more, wouldn't it? Like you, I didn't know I had had the mesh until I started having problems with it, and went back….I suppose this time it was the timing of all the publicity, and all the rest of it*. (PROSPECT Participant 1 focus group 1)

However, there were also opposing views within the PROSPECT group. One participant ranked ‘Summary of overall trial results’ as the most important information, citing that to know or make sense where they fitted in, she needed to know the overall results of the trial.

*To me, you need to know what the overall trial results are to know where you fitted in…. I do think that the individual thing is very important, yeah…. So, I put [it] second…, they are partially the same thing*. (PROSPECT Participant 1 focus group 1)

The VUE participants ranked ‘Summary of overall trial results’ overall as the most important information content item, while this item was ranked sixth by the PROSPECT participants. The reasons why a summary of overall trial results was ranked most important by some of the VUE participants included reassurance that they had received the best treatment and being able to compare success rates between the different treatments.

*Just how beneficial, how beneficial each type of surgery is basically looking back….I suppose reassurance what you had done was the right thing, but I'm sure you know, it definitely was*. (VUE Participant 2)

*Well obviously, namely how many people it was successful in, and I suppose how many people were in it in the first place, how many people it was successful, that would be an idea….Aye, well really just to find out the percentage of success and the percentage of non‐success, shall we say?* (VUE Participant 6)

### 3.3 Other information content items ranked highly

The information content item *‘*Information about which treatment was received in the study’ was ranked highly by both participant groups. It ranked as the second most important for the PROSPECT participants and third most important for the VUE participants. Much of the decision‐making for why this information content item ranked highly was that this information would enable participants to set the results in context when receiving the results of the trial.

*Yes. That would be good to know. If you couldn't remember or you'd forgotten, because it has been some time, that would be really good to know: a reminder, and this is what happened here, and this is what you had done…[so when reading results]… It makes sense…. And there's clarity*. (VUE Participant 3)

Another information content item that consistently ranked high in both groups was ‘How to report side effects (if you are still experiencing these)’. It ranked the second most important information content item with the VUE participants and third most important with the PROSPECT participants. For many participants, the reason it ranked so highly was that it would enable them to have contact details if they were still experiencing problem, again to receive reassurance.

*I think it's like anything, you're not too sure what side‐effects. Is it a normal thing to have or is it just you? Or is it because of that, so… you're unsure, I tend to go, ‘Oh, it'll pass, it'll go’, so it's because of that grey area of being never sure what would be a side‐effect, you know?*. (VUE Participant 4)

### 3.4 Information content items deemed least important

The lowest‐ranked information content item across both participant groups was ‘A thank you for your contribution to the study’. A number of reasons for this were voiced by both the VUE and PROSPECT participants including altruistic reasons (such as helping others) and, because they had been thanked throughout their participation in the trial, many felt that a final ‘thank you’ was less important.

*Well, the National Health Service is so good for us, isn't it? We shouldn't need a thank you for doing something so simple as answering a few questions…*. (VUE Participant 5)

*We are doing it because we want to do it…. to help with other people*. (PROSPECT Participant 3 focus group 1)

*Because I feel that you're thanked at the very beginning for starting the study. We're thanked along the journey. Every letter, every questionnaire that you see, there's a thank you and it doesn't have to be a verbal thank you. You know, it's documented down. Each letter comes with a thank you and I think, for me, as a level of appreciation to the team, because I thank them because they've made me better. It goes both ways*. (VUE Participant 3)

### 3.5 Preferred mode of delivery

The results from the ranking on the preference of how participants would want to receive results are presented in Table 3. Delivery of results ‘by post’ was the preferred mode of receiving trial results across both participant groups. A strong desire to receive a hard copy of results for something to refer back to and to keep track of treatments was voiced by many of the participants when exploring the decision‐making for why this mode of delivery ranked so highly.

**Table 3:** Ranking of preferred mode of delivery

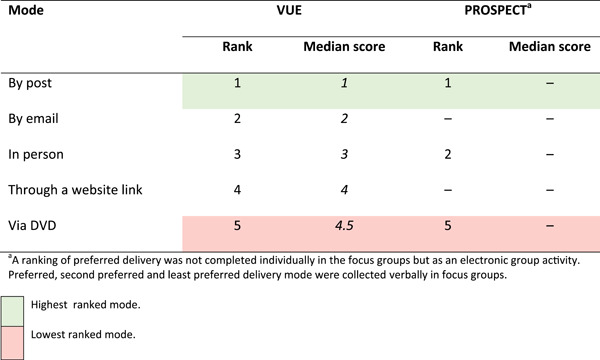

^a^A ranking of preferred delivery was not completed individually in the focus groups but as an electronic group activity. The preferred, second preferred and least preferred delivery modes were collected verbally in focus groups

*Yes, I do. I keep everything. I'm not a hoarder, but I do like to keep… If it's something that's happened to me or it's something that important.….I do hold onto everything. I like to have some kind of reference, so if anything was to go wrong with my health in the future…* (VUE Participant 3)

‘In‐person’ dissemination was ranked as the second most preferred method by the PROSPECT participants and third most preferred method by the VUE participants. When this was explored in further discussions, it was expressed by a number of the participants in both groups that there was interest in this method of dissemination, with many liking the idea of having someone to talk to and being in a group setting to be able to share their experiences and gain reassurance regarding surgical outcomes. The reasons given for not ranking ‘in person’ dissemination of results higher in some cases included a concern of others and one's own availability to come to a meeting.

*Could it have been helpful, rather than having it in a newsletter, that you got the opportunity to talk to somebody—so either had a meeting like this where the researchers were here, and they could say, this is the study, and these were the results, and then it would give you the opportunity to have (to speak to others to know)…. you are not the only one it's happening to*. (PROSPECT Participant 4 focus group 2)

*Yes, because I thought… it would be nice to see someone face to face, I like to do lots of things face to face, I want to order something, I prefer to talk to somebody. That's just a preference but it's not always possible, for time, for both parties. Yeah, that's the reason why*. (VUE Participant 4)

Additionally, one participant also voiced a strong desire to still receive a hard copy of the results to have the results ‘in black and white,’ and this would still be the preference if an in‐person format was offered.

*I suppose I would prefer it, as you say, in black and white. And I mean, if they wanted to have a discussion after you read this, that would be acceptable. But I'd like to have a (copy) to begin with*. (VUE Participant 6)

### 3.6 Least preferred mode of delivery

The lowest‐ranked mode of dissemination was by *‘*DVD’ in both participant groups. A reason voiced for this was individuals' abilities with technology or not having access to it. This theme of abilities and accessibility in relation to technology continued when comparing the other methods of result delivery in the ranking exercise to a postal method such as a website link or email.

*Aye. DVD? The thing is, I'm not very good with technology, you see. I do use email and… but I don't like it!*. (VUE Participant 5)

*I'm afraid I don't have a computer. I don't want one.I'm happy reading a book, and watching the television*. (PROSPECT Participant 5 focus group 2)

*Yes. I do have an iPad, but I struggle. Yes, definitely….Not so up to date with all these things…*. (VUE Participant 1)

### 3.7 Receiving results

Both groups of participants were also asked if they could think of any situations in which they may not want to receive trial results. There was a strong overall consensus in both groups that participants should be provided with the results, with one participant then suggesting that the onus would then be on the participant to decide whether or not to read the results.

*At the end of the day if you've taken part in it you can choose not to read it, but maybe everybody should just be sent them anyway because there might be a time that you want to look back…* (VUE Participant 2)

## 4 DISCUSSION

Our findings provide insight into what information trial participants deem as important and how they would like trial results delivered. A mixed‐methods approach allowed us to explore why trial participants deemed certain items important and why certain modes of delivery were preferred. Participants felt that it was vital to know and receive the results of the trial they took part in, which is consistent with other studies.^1^, ^8^, ^12^, ^25^, ^26^ There was, however, discordance between and within participant groups about which information content item was the most important. Overall, the PROSPECT participants (who had previously received trial results) reported being able to receive their *individual results* as the most important content item because of a sense that their own experiences did not align with the overall trial findings. VUE participants (who had not received results) ranked *overall trial results* as the most important information content for reassurance and to allow comparison between treatments. Similarly, participants may not always be sure what information they want to receive until they are provided with additional information; in other words, people do not know what they do not know. This may explain the discordance between what participants initially thought was important information to receive in trial results and what they then ranked as important when presented with a list of items.

Both participant groups ranked *a thank you* as the least important content item. It is possible that our study cohort felt that that they had already been thanked and acknowledged throughout their trial participation. A recent study found that participants felt that receiving results acknowledged their own individual contribution.^27^ It is unclear if this was also the case in our study, although ‘wanting to help other people’ was identified as a key theme for the *thank you* item ranking the lowest. These findings align with previous research showing that altruistic reasons were often voiced by would‐be trial participants as reasons for clinical trial participation.^28^, ^29^

Comparison of trial participant groups suggests that the experiences of trial participation may affect the information content that participants wish to know. Seeking reassurance from the trial results was a clear theme that contributed to the higher ranking of knowing which treatment was received and how to report side effects. In a previous survey of 3381 research participants, 70.6% wanted to have results that were specific to them.^15^ Previous qualitative research has also shown that participants wish to receive information about the treatment that they received to better understand ongoing health issues and seek advice appropriately.^16^, ^17^ Differences between the PROSPECT and VUE participants' rankings may be attributable to the timing of our study in relation to the host trials and results received, that is, PROSPECT participants had already received the 2‐year trial results, whilst VUE participants had not. However, these differences in whether participants had received trial result summaries or not were only considered post data analysis and as such not prospectively included for investigation. These differences in ranking may also be due to the difference in data collection methods for each cohort, that is, focus group versus individual interviews.

In both groups, the preferred mode of delivery of the trial results was *by post*, with reasons including having a *hard copy* to be able to refer back to and it being a more accessible mode of dissemination compared to those that required the use of and access to technology. These findings support previous work in this area, which found that participants wished to receive a hard copy of the results.^27^, ^30^ Having the results in an accessible format was a running subtheme and this finding may also relate to the older age of our study cohort. This was also highlighted in previous qualitative work that considered mode appropriateness according to the demographic, suggesting that older participants may not have access to certain modes of communication.^12^, ^25^

Although the HRA guidance does not currently suggest ‘in‐person’ as a mode of delivery for dissemination, there appeared to be an interest in this option due to having someone to talk to and to share experiences with.^6^ Previous work in this area reports similar findings.^31^, ^32^ In‐person delivery of trial results may be geographically challenging in multicentre randomized‐controlled trials. However, holding events online may overcome some of these challenges and could be an area explored by trial teams for future ‘in‐person’ activities, but recognition of problems with digital access would also need to be considered and balanced.^33^ Our findings suggested that participants may wish to receive the results in more than one format. Previous research suggests that results could be made available to participants in a variety of ways rather than just providing one mode of delivery.^25^ Working with patient partners to design the content and mode of delivery of trial results summaries will help to ensure that preferences are built into trial dissemination activities.

Future research priorities have been identified in this area through a priority‐setting partnership focusing on methodological priorities for patient and public involvement in clinical trials.^34^ The question of how patient and public partners are involved in dissemination of trial results and the assessment of effectiveness was identified as the eighth most important priority (out of 42) in a stakeholder consensus process.^33^ Research to address this gap is needed alongside more in‐depth explorations of experiences of receiving results across a range of trial settings. In addition, randomized evaluations to determine which methods are most effective could also add value, but determining the most appropriate outcomes to measure to determine whether this has been done well also warrants attention. All of these research activities should ensure that they include patients and the public as partners in the research process.

## 5 STRENGTHS AND LIMITATIONS

A purposeful form of data sampling (rather than convenience sampling) may have provided a greater spread of opinions and views; however, we were limited to geographic location and to trial participants who had consented to be contacted about other related research. Another potential limitation of this study was the size of the sample (10 participants in two focus groups and 6 participant interviews). Participants were recruited from two trials in a similar disease area and patient population (gender and age); therefore, our findings may not be generalizable. Demographic data were not collected, which may relate to participants' views and opinions expressed in the focus groups and interviews.

In addition, the preferred mode of dissemination could be related to the age group included in this study and could be a potential limitation of our study. A study by Purvis et al.^25^ identified that the preferred mode of delivery of research results did appear to vary depending on the age of the respondent. Younger participants expressed an ‘openness’ to receiving findings via email, text or other social media, while older participants preferred standard post or face‐to‐face communication.

A mixed‐methods approach enabled greater insight into what participants deemed as important. Although previous research has found that the group dynamic in focus groups may increase the extent of group agreeableness,^35^ VUE participants were interviewed individually and would not have been influenced by this. Our study participants had participated in a real randomized‐controlled surgical trial rather than a hypothetical or oncology‐based trial. Thus, these findings have the potential to be more informative to trial researchers from wider trial populations for consideration in their dissemination plans.

## 6 CONCLUSION

Our findings provide insight into what information trial participants deem as important when receiving trial results and how they would like trial results delivered. Participants feel that they should be able to access trial results and receive them in an appropriate format. The HRA and other regulatory research stakeholders should implement systems to ensure that dissemination of results to trial participants becomes common place and includes patient partners in the development of such information. Future research could explore feedback development from an early stage using a consultative approach in other trial settings and patient demographics through embedded research within clinical trials. As highlighted in the Limitations section, this study included a small sample of women from a similar disease area; therefore, the results may not be applicable to other populations and disease areas. We acknowledge that further research in participants with a range of characteristics (including for e.g., gender, age, ethnicity) and disease areas is required to determine appropriate modes of delivery across different cohorts.

## CONFLICT OF INTERESTS

The authors declare that there are no conflicts of interest.

## AUTHOR CONTRIBUTIONS

Jessica Wood, Seonaidh C. Cotton and Katie Gillies designed the study. Jessica Wood conducted the VUE interviews, Katie Gillies and Seonaidh C. Cotton led the focus groups and Katie Gillies and Jessica Wood conducted the analysis. Jessica Wood wrote the original draft of the manuscript. All authors contributed to writing and critical appraisal of the manuscript.

## ETHICS STATEMENT

Ethical approval was sought and granted by the College of Life Sciences and Medicine Ethics Review Board CERB/2017/7/1491 on 14 August 2017.

## Data Availability

Consent was not received to share original data sets and, therefore, research data are not shared.

## APPENDIX 1

**FOCUS GROUP TOPIC GUIDE**

**Introductions**

Thank everyone for coming and introduce yourself, distribute consent forms for completion.

Once seated and commencing the audio‐recording, ask each person to introduce themselves by their first name.

**Setting context**

The purpose of today's focus group is to identify what key information participants want to have available in a results summary of a Phase III trial that they have taken part in.

Explain what is meant by a Phase III trial; these are trials run to assess the effectiveness of the new intervention and typically have 300–3000 participants taking part. They aim to determine how effective a drug or surgical intervention is compared to another drug or intervention and in some trials more than two treatments are compared. For example, in PROSPECT, it was actually two randomized‐controlled trials comparing standard (native tissue) repair alone with standard repair augmented with either a synthetic mesh (the mesh trial) or a biological graft (the graft trial) for prolapse surgery in women.

**During the focus group**

Start off by asking participants to talk about their experience of participating in PROSPECT.

- Do you remember receiving the results of the trial?
- What stood out for you from the results/what do you remember?
- Was there anything else you wanted to know; if yes, where from?
- Was the information presented in a way that was understandable?

‐ If yes what was helpful/if not, what was not clear?

- We sent the results by post in the form of a newsletter, how did you find this? Would you have liked a link to more information? Would you have preferred it delivered differently, for example, face to face or the internet?

**Interactive exercise**

We are now going to complete an interactive exercise; some questions require you to rank items and others are yes/no questions. Introduction to the ranking exercise: It is a bit like voting in the local elections when you need to rank your first choice of candidate, your second choice of candidate and so on. Rather than using a voting slip, we will be using some handheld devices to complete this.

For the warmup exercise: First, can everyone chose their favourite destination by selecting A, B or C on your device. OK—has everyone done this now. Now select your second favourite destination. Then, the least favourite.

**Following the exercise**

If someone would have liked results in person, prompt to find out who they would like to receive the results from and in what setting: doctor (1 on 1), nurse, researcher at a patient coffee morning and so forth.

For any discrepancies in order of importance (example two items have been given same waiting) for Question 1, use the printed 10 individual items to explore with the group if on review they would change their responses and why.

**Second part of focus group**

- Did receiving the results of PROSPECT make you feel that you were part of the research and that your input/time was recognized, why/why not?
- Based on your experience in PROSPECT and receiving the results of the study, would you take part in future research and what has influenced your opinion on this?
- Can you think of any situations where you would not want to receive the results? Would you have liked a choice about receiving the results?

- Was there anything else you would like to add?
  If discussions veer into PROSPECT‐specific concerns, highlight that the purpose of the focus group is about trial feedback in general and not to provide any further information about the PROSPECT trial.
- Closing the focus group and reflecting the PROSPECT results

Now that you have seen the PROSPECT results newsletter again today and following our discussions today, was it how you remembered? Was there anything that you had not remembered about the results?

Thank the participants for their time. Confirm that the statements and comments made today will be anonymized (i.e., study numbers and not names of individuals will be used in the interpreting of the data). Ask them if they would like to receive the results of this project and in what format.

**Interview topic guide**

- Can you tell me a little about your experiences of participating in VUE?
- What do you understand about clinical trials?

There are different types of clinical trials. Some test new medicines or vaccines, some look at new combinations of existing treatments or look at delivering an existing treatment in a different way and whether it will make it more effective or reduce any side effects. Drug trials are probably the most familiar type of trial for many people, but clinical trials are not always about just testing medicines. They can be used to test other ‘interventions’ aimed at changing a person's behaviour or lifestyle (for exercise habits). Trials of different surgical procedures are not as common as drug trials. Surgical trials may, for example, compare different types of operations, or they may compare surgery with a nonsurgical treatment.

In VUE, they compared different operations for prolapse in women with or without a womb. There were two studies within VUE.

‐ Uterine trial (women with womb): Vaginal hysterectomy compared with an operation to suspend the uterus without removing it, and
‐ Vault trial: Suspending the vault from below (the vaginal route) compared with suspending it via the abdomen (tummy).

For the purpose of today, we are not going to be discussing the VUE results, but I would like to find out about your experiences of taking part in a trial like VUE and your views on receiving results from trials like VUE.

- What do you remember being told when you consented to take part in VUE?
- If they do not mention results—ask them if they remember that.
- What kind of information would you like to see in a results summary? You will need some prompts here as example items—what were the overall findings? link to published paper? What to do if you have questions? (use ‘What else’ as a continuous prompt).
- What way would you like it delivered? Some examples include by post, email, podcast (video of the researcher explaining the results) or meeting with the researcher.

Now, we are going to complete a set of ranking activities; the first is based on information you we expect to receive in a trial results summary or newsletter. Could you order these items based on what you think is the most or the least important?

Time to allow participant to complete Question 1.

Discuss how these items compare to what the participant has identified as important based on earlier discussions.

Ask the participant why he or she chose that item as the most important.

Ask the participant why he or she chose that item as the least important.

Now, I would like you to complete another activity exercise based on the most to the least preferred way of receiving trial results.

Time to allow participant to complete Question 2

Now that we have looked into what information you would want to be included in trial results and how you would want it delivered, can you think of any situations where you might not want to receive the results for study? For example, what about in cases of bereavement? Should the family still find out the results of the trial?

Do you think it is important that you are asked when you first agree to take part in a study if you would like to receive the results and how you would want it delivered to you?

This study is all about receiving results ‐would you like to receive the results from this student project? Also, how would you like it delivered to you?

Finally, the information provided today will be used by the VUE team to better prepare the trial results summary for participants. This will be sent out in Spring next year.
